# One Health genomic surveillance identified high-risk carbapenem-resistant ST821 clones of Acinetobacter baumannii in Nigerian clinical and community settings

**DOI:** 10.1099/mgen.0.001768

**Published:** 2026-06-29

**Authors:** Khalifa J. Saleh, Oliver McNeilly, Hafsat M. Tsangaya, Maryam I. Musa, Samantha Palethorpe, Nafisat A. Kachalla, Husna F. Ibrahim, Jeremy S. Brown, Hamisu M. Salihu, Richard A. Stabler, Ibrahim Yusuf

**Affiliations:** 1 Department of Microbiology, Faculty of Life Sciences, Bayero University Kano, Kano, Nigeria; 2 Department of Microbiology, Federal University Dutsin-Ma, Kilometer 60, Along Katsina-Kankara Road, Katsina, Nigeria; 3 Department of Infection Biology, London School of Hygiene and Tropical Medicine, London, UK; 4 Department of Microbiology, Faculty of Life Sciences, Ahmadu Bello University, Zaria, Nigeria; 5 UCL Respiratory, University College London, London, UK; 6 Department of Microbiology, Faculty of Life Sciences, Modibbo Adama University, Yola, Adamawa, Nigeria; 7 Department of Medical Microbiology and Parasitology, Faculty of Basic Clinical Science, College of Health Sciences, Bayero University Kano, Kano, Nigeria; 8 Department of Microbiology and AMR Research, Kano Independent Research Centre Trust (KIRCT), KM1. Kwanar Dawaki, Kano, Nigeria; 9 Department of Epidemiology and Population Health, Kano Independent Research Centre Trust (KIRCT), KM1. Kwanar Dawaki, Kano, Nigeria

**Keywords:** *Acinetobacter baumannii*, antibiotic resistance, genomic surveillance, One Health, Nigeria, Multidrug resistance

## Abstract

*Acinetobacter baumannii* is a Gram-negative opportunistic pathogen increasingly implicated in severe hospital and community-acquired infections. In Nigeria, widespread antibiotic use across human, animal and environmental sectors contributes to antibiotic selection pressures that drive the emergence and spread of high-risk multidrug-resistant lineages. This One Health genomic surveillance study investigated the prevalence, resistance mechanisms, genomic characteristics and reservoirs of * A. baumannii* across multiple sources in Kano, Nigeria. A total of 3,235 samples were collected from clinical samples, hospital environments, non-hospital environments and poultry droppings. *A. baumannii* isolates were identified by growth on MacConkey/CHROMagar and confirmed by *bla*_OXA-51-like_ amplification. Antimicrobial susceptibility was assessed using the Kirby–Bauer disc diffusion method. Of all samples, 62 isolates were confirmed as *A. baumannii*, originating from clinical samples (*n*=24), hospital environments (*n*=33), non-hospital environments (*n*=2) and poultry droppings (*n*=3). Whole-genome sequencing of 27 select isolates was performed to characterize resistance and virulence genes, sequence types (STs), mobile genetic elements, genomic islands and SNP-based relatedness. Thirteen *bla*_OXA-51-like_ and one *bla*_OXA-58-like_ variants were detected. MLST revealed 21 STs using the Pasteur scheme and 14 using the Oxford scheme, many of which were susceptible to most antibiotics tested. In contrast, isolates N9, N13 and N16 (ST821) and N19 (ST625) were resistant to all tested antibiotics, including carbapenems. The highly resistant ST821 clones were isolated from a municipal dumpsite, hospital floors and a hospital bedside surface and separated by only 5–17 SNPs, indicating close relatedness and clonality. Previously reported ST821 strains from the UK, Pakistan, Germany and Nigeria were highly susceptible to antimicrobials. Although no plasmids were detected, complementary genomic islands were identified in N9, N13 and N16 containing *bla_NDM-1_*, *tetA/tetR*, *folP*, *aac*, *aphA*, *dinB*, *cueR* and *trxC* and therefore likely to confer resistance to antimicrobials. In conclusion, this study provides the first broad One Health genomic overview of *A. baumannii* circulating across human, environmental and poultry sources in northern Nigeria, revealing substantial genomic diversity and the identification of a closely related extreme drug-resistant ST821 lineage spanning hospital and community environments.

**Impact Statement**

This study presents a One Health genomic assessment of *Acinetobacter baumannii* in Nigeria using isolates obtained from hospital environments, community sites and animal-associated sources. Through large-scale sampling supported by whole-genome sequencing, we describe the ecological distribution and genetic diversity of *A. baumannii* circulating in Kano, northern Nigeria. The findings indicate that hospital environments remain an important reservoir, while also highlighting the spread of a locally expanded extensive-drug-resistant ST821 lineage carrying *bla*_NDM-1_ and chromosomally integrated resistance islands beyond the hospital setting.

These data are important for clinical practice and public health. The recovery of resistant strains from hospital surfaces supports the need for stronger infection prevention measures, including regular cleaning of high-touch areas, improved hand hygiene, equipment decontamination and closer surveillance in high-risk units. Detection of related strains in community environments also points to the importance of safe waste management and better disposal systems to reduce environmental persistence and onward spread.

Our findings further reinforce the need for antimicrobial stewardship guidelines that promote appropriate antibiotic use and culture-guided treatment. More broadly, this study helps address the limited genomic data available from sub-Saharan Africa and highlights the value of integrated surveillance in identifying and containing high-risk *A. baumannii* lineages before wider regional or international spread occurs.

## Data Summary

Whole-genome sequencing data are available under BioProject PRJNA1367586. NCBI accession numbers for the FASTQ data are SRR37101830–SRR37101854 (details in Supplementary Data File 1).

## Introduction

*Acinetobacter baumannii* is a clinically relevant, opportunistic Gram-negative coccobacillus bacterium that has emerged as a leading cause of severe healthcare-associated infections due to its environmental resilience and capacity for multidrug resistance (MDR) acquisition [1]. It has emerged as a globally significant nosocomial pathogen, causing a range of serious infections such as pneumonia, bloodstream infections, wound infections, urinary tract infections and meningitis, particularly in critically ill or immunocompromised patients [2]. Its clinical success is linked to its remarkable ability to withstand desiccation, persist on inanimate surfaces for prolonged periods and acquire antimicrobial resistance genes (ARGs) through horizontal gene transfer and mutation [3]. In recognition of its public health threat, the World Health Organization recently listed carbapenem-resistant *A. baumannii* as the second global bacterial priority pathogen for urgent research and development of new antibiotics [4]. Although *A. baumannii* predominantly causes healthcare-associated infections [5], a number of recent studies have also reported community-acquired *A. baumannii* pneumonia, bloodstream and skin/soft tissue infections, usually associated with comorbidities. *A. baumannii* has been isolated from a diverse range of environmental and animal sources, including sewage, hospital wastewater, soil and animal production facilities [6,8]. It is speculated that these extra-hospital reservoirs could contribute to the increasing number of community-acquired *A. baumannii* infections [9].

In Nigeria, *A. baumannii* is under-reported in hospital microbiology laboratories, with hospital data indicating low detection rates and a heterogeneous resistance profile among reported cases [10,12]. In Kano State specifically, studies from tertiary hospitals (including Aminu Kano Teaching Hospital (AKTH), Murtala Muhammad Specialist Hospital (MMSH) and Muhammad Abdullahi Wase Specialist Hospital) have reported *A. baumannii* isolation rates of ~6.5–10.1% from clinical samples such as wound swabs, urine and respiratory specimens, often in patients with prolonged hospital stays [13]. High rates of multidrug-resistant strains (up to 85.7%) are reported in isolates from these facilities [13]. Similarly, environmental surveillance in Kano reveals a concerning 15.7% prevalence of *A. baumannii* in both hospital surfaces (beds, chairs and drawers) and non-hospital settings (student hostels, soil and sullage), which may indicate potential reservoirs that facilitate ongoing transmission between clinical and environmental sources [12].

The isolates were recovered from bloodstream infections, wounds, respiratory specimens, urine and ICU patients, frequently with MDR phenotypes. For instance, a multicentre genomic study of CRAB recovered from Nigerian hospitals in the southwest region between 2016 and 2020 revealed marked genetic diversity and circulation of multiple resistant lineages [14]. In addition, a prospective ICU study also from southwest Nigeria documented rectal colonization and probable nosocomial transmission of CRAB within local hospitals [15]. Reports from Kano State have similarly shown substantial recovery of *A. baumannii* from both clinical and environmental sources, including a high prevalence of CRAB [12].

MLST, a robust technique for defining the clonal structure and evolutionary trajectories of bacteria and enabling the differentiation of high-risk international clones (ICs) from emerging lineages [16], was used in this study. Unlike the MDR lineages that dominate clinical settings, ST821 has been previously recovered from diverse One Health sources that include humans and environmental reservoirs from the UK, Pakistan, Germany and Southwest Nigeria [10]. A distinguishing feature of ST821 isolates is their notable susceptibility to standard antimicrobial agents, suggesting that they may represent a significant yet less-pressured strain [5]. Against this background, the identification of ST821 clones is particularly concerning, as it suggests that this clinically adapted lineage can disseminate throughout Nigerian and potentially global healthcare settings. Reports on environmental and animal reservoirs are even scarcer. Recently, novel and rare variants (*bla*_OXA-1328_ and *bla*_OXA-707_) were isolated from Nigerian community sewage and soil, respectively [17], highlighting the presence of diverse environmental reservoirs. Whether these sources represent true reservoirs of *A. baumannii* in Nigeria remains unclear, which necessitates the need for further investigation.

One of the major drivers of antimicrobial resistance (AMR) in Nigeria is the widespread use of antibiotics in both humans and animals, particularly in poultry production, where antimicrobials are routinely added to feed or water for growth promotion and prophylaxis [18]. This practice can exert strong selection pressures on poultry gut bacteria and eventual evolution of resistant bacteria in the intestinal tract, which are then shed into the environment through droppings. These droppings are often applied directly to farmlands as manure, which can lead to contamination of soil, surface water and crops with resistant bacteria and residual antibiotics. The potential environmental transmission of resistant bacteria in Nigeria can be further compounded by the indiscriminate disposal of untreated or partially treated effluents from healthcare facilities, abattoirs, tanneries and pharmaceutical plants into the environment [19]. Biomedical waste from some hospitals ends up in open municipal dumpsites or landfills located near residential areas, which can facilitate environmental exposure to resistant organisms [20]. Despite these risks, there are limited data on hospital and extra-hospital sites which could be sources of *A. baumannii* infection in Nigeria.

This knowledge gap limits our understanding of the epidemiology, reservoirs and cross-sectoral transmission dynamics of * A. baumannii* in Nigeria. Therefore, in this study, we aimed to determine the prevalence, antimicrobial susceptibility profiles and the potential reservoirs of *A. baumannii* in human, poultry, clinical and non-clinical environmental sources.

## Methods

### Study sites

A One Health cross-sectional surveillance study was conducted from March 2023 to January 2025 in Kano, Nigeria, to collect and isolate *A. baumannii* from human, animal and environmental samples. Clinical samples were collected from patients visiting four major hospitals: AKTH, MMSH, Muhammad Abdullahi Wase Teaching Hospital (MAWTH) and Infectious Diseases Hospital (IDH). AKTH is a 500-bed federal tertiary institution, serving as the primary referral hub for the entire North-West geo-political zone. MMSH is a specialist hospital in West Africa, with an estimated 7,000 outpatient visits daily and manages up to 1,000 deliveries monthly. MAWTH is a state-owned teaching hospital with 250 beds, bridging the gap between specialized research and routine state-level clinical care. IDH is a public specialized secondary healthcare facility which serves as a critical centre for managing infectious diseases.

The four hospitals included in the study are among the major public referral and specialist healthcare facilities in Kano State, serving large and heterogeneous patient populations from both urban Kano and surrounding northern Nigerian states. These facilities were selected as they manage high patient volumes, frequent antimicrobial usage, prolonged admissions and severe infections, all of which are recognized risk factors for *A. baumannii* colonization and infection [13]. Although Kano shares common features with many Nigerian urban settings, the purposive design may limit how directly the findings can be generalized nationally.

Environmental samples were collected both within and outside hospital settings, including hospital high-touch surfaces, as well as non-hospital sites. Environmental sampling sites were selected to represent locations with frequent human contact and potential bacterial persistence, including hospital high-touch surfaces, community waste disposal areas, soil, wastewater and student residential settings. Poultry farms (rearing broiler and layer chickens) and the Janguza live bird market were included, as poultry production and produce marketing are major agricultural activities in Kano, involving regular antimicrobial use, dense animal populations and close contact between humans, animals and the environment, creating opportunities for bacterial contamination.

### Sample collection

#### Clinical samples

Early-morning, midstream urine samples were collected under aseptic conditions. Collected specimens were immediately capped, labelled with patient identifiers and transported to the laboratory within 2 h of collection [21]. Early-morning sputum specimens were collected aseptically from patients attending the study hospitals in Kano State. Patients were instructed to collect sputum samples aseptically from early-morning deep coughs into a labelled sterile, leak-proof, wide-mouthed container, which was taken to the laboratory for analysis [22]. To collect samples from wounds, the wound site was thoroughly cleansed with sterile 0.9% normal saline to remove superficial contaminants and necrotic debris. A sterile swab was then rotated over a 1 cm² area of viable tissue at the centre of the wound, with sufficient pressure applied to express wound exudate from the underlying tissue layers. The swab was immediately placed into Amies transport medium, labelled and transferred to the microbiology laboratory within 2 h [23].

#### Environmental samples

Sterile SARSTEDT transport swabs were used for surface sampling. Hospital surfaces from wards were swabbed as follows: a standardized area of about 10 cm×10 cm was swabbed using a systematic technique of horizontal, then vertical strokes with firm pressure to ensure adequate collection of micro-organisms [24]. Surfaces of wastes from designated waste collection points within the hospitals and communities were swabbed aseptically. This includes used culture dishes, bandages, cotton and transfusion kits (biomedical wastes) and various waste materials that include plastics, polythene bags and food waste (community dumpsites). Additionally, swabs were taken from the exterior surfaces of incinerators used for waste disposal in the hospitals, like those taken from hospital surfaces. Swabs were immediately placed back and kept on ice for transportation to the laboratory [17].

#### Poultry dropping

Freshly voided droppings were collected immediately upon excretion. Samples were taken from the centre of the faecal dropping using a sterile spatula to minimize environmental surface bacteria. The spatula was placed into sterile conical tubes containing transport medium and kept on ice for transportation to the laboratory [25,26].

### Microbiology

#### Isolation

Sputum, urine and wound swab samples were inoculated directly onto MacConkey agar using a sterile wire loop. The inoculated plates were then incubated aerobically at 37 °C for 24 h. Environmental swabs, soil and chicken dropping samples were enriched in mineral salt medium (MSM)+0.2% acetate. The MSM was prepared in house by dissolving 10 g of KH_2_PO_4_, 5 g of Na_2_HPO_4_, 2 g of (NH_4_)_2_SO_4_, 0.2 g of MgSO_4_.7H_2_O, 0.001 g of CaCl_2_ (2H_2_O) and 0.001 g of FeSO_4_.7H_2_O in 1 l of distilled water, autoclaved at 121 °C for 15 min, after which 0.2% acetate was added [8]. Enrichment was carried out by adding 1 g of soil and dropping samples each to 100 ml MSM/acetate and 5 ml into the swab stick container and incubated at 37 °C for 6 h. One millilitre (1 ml) was then plated onto MacConkey agar and incubated at 37 °C for 24 h [8]. For air samples, a passive settle plate method was employed using MacConkey agar by exposing plates for 30 min to collect air from hospital wards and waste disposal sites [27]. All samples were transported at 4 °C to the AKTH Microbiology laboratory for analysis. Where the analysis could not be completed immediately, samples were stored in a refrigerator at 4 °C.

#### *A. baumannii* identification

Presumptive *A. baumannii* colonies were identified via morphology (non-lactose fermenting, shiny and tiny with raised centres), Gram staining (Gram-negative) and biochemical tests (oxidase, citrate utilization, triple sugar, urease and motility tests) as previously described [12]. Selected colonies were sub-cultured on CHROMagar Acinetobacter (CHROMagar, France) to differentiate *Acinetobacter* species from other Gram-negative bacteria [8,28]. *A. baumannii* isolates were confirmed by amplification of *bla*_OXA-51-like_ genes as previously described (Table 1) [8,29, 30].

**Table 1.** Distribution of *A. baumannii* from human, animal and environmental sources

Isolate source	No. of collected	No. of positive for bla_OXA-51-like_ (%)
Clinical	580	24 (4.1%)
Hospital environment	1,605	33 (2.0%)
Non-hospital environment	530	2 (0.3%)
Poultry	520	3 (0.5%)
Total	3,235	62 (1.9%)

Isolated *A. baumannii* colonies were suspended in 200 µl nuclease-free water, and genomic DNA was extracted by heat lysis in a 95 °C water bath for 10 min. The lysate was then centrifuged at 10,000 ***g*** for 5 min, and the supernatant was used as template DNA. PCR amplification of species-diagnostic *bla*_OXA-51-like_ gene used previously described primers (for 5′-TAATGCTTTGATCGGCCTTG, Rev 5′-TGGATTGCACTTCATCTTGG) [31].

The following thermal cycler conditions were used: initial denaturation at 95 °C for 5 min; 35× cycles of denaturation at 95 °C for 30 s, annealing at 53 °C for 30 s and extension at 72 °C for 30 s; final extension at 72 °C for 7 min. PCR products were then visualized via agarose gel electrophoresis to identify expected 353 bp *bla*_OXA-51_ amplicons [8].

#### Antimicrobial susceptibility testing

Antimicrobial susceptibility testing was performed using the Kirby–Bauer disc diffusion method in line with locally available laboratory capacity. The isolates’ susceptibilities to ampicillin–sulbactam (10 µg Oxoid), cefepime (30 µg Oxoid), ceftazidime (30 µg Oxoid), ceftriaxone (30 µg Oxoid), ciprofloxacin (5 µg Oxoid), gentamicin (5 µg Oxoid), imipenem (10 µg Oxoid), levofloxacin (5 µg Oxoid), meropenem (10 µg Oxoid), piperacillin–tazobactam (10 µg Oxoid), sulfamethoxazole–trimethoprim (10 µg Oxoid) and tetracycline (30 µg Oxoid) were determined. Results were interpreted according to CLSI M100, 33rd edition (2023) guidelines, using breakpoints applicable to *Acinetobacter* spp. where available [32] (File S1, available in the online Supplementary Material).

### Whole-genome sequencing

Genomic DNA was isolated using Promega Wizard Genomic DNA Purification Kit following the manufacturer’s instructions. Quality was determined using Nanodrop. Whole-genome sequencing (WGS) was carried out by Novogene (UK) using NovaSeq X Plus PE150 platform (Illumina, USA). Raw reads (paired-end 2×150 bp) were subjected to quality trimming using TrimmomaticPE v0.39 using the following parameters: Leading: 3, Trailing: 3, Sliding Window: 4:20 and Minlen: 36. Read quality was further assessed using FastQC v0.12.1. Trimmed reads were aligned to the complete genome of *A. baumannii* strain MDR-TJ (NCBI Acc. No. CP003500.1) using BWA MEM with default parameters, and identification of SNPs and indels was performed using GATK version, and the alignments and SNP calls were visualized in ACT (Artemis). *De novo* sequence assembly was performed using Unicycler v0.5.1 with both default settings and the following modified settings: minimum fasta length=500, minimum k-mer fraction=0.4 and maximum k-mer size=301.

Assembly metrics were assessed with QUAST v5.2.0. Contig ordering was carried out against the reference genome CP003500.1 using ABACAS v1.3.1 with the command option -dmbc, and any unmatched contigs were appended to the ordered ones. Genome completeness and contamination were assessed for each assembly using CheckM v1.2.4 using an *Acinetobacter*-specific taxonomy marker set.

Gene annotation was performed using Prokka v1.14.6 with settings optimized for Gram-negative bacteria. Assembled contigs with unusual genome lengths were taxonomically classified using Kraken2 and the minikraken_8 Gb_20200312 database, and sequences with high identity calls other than *A. baumannii* were excluded from further downstream analysis [33].

To identify AMR genes, assembled genomes were screened using ABRicate v1.0.1 against the CARD and NCBI AMRFinderPlus databases with a minimum coverage set to 95%. MLST using the Pasteur and Oxford scheme was conducted with the MLST software v2.23.0. *In silico* serotyping of capsule (KL) and oligosaccharide (OCL) loci was done with Kaptive v2.0 using *A. baumannii* reference databases [34, 35]. Core genome MLST profiles were generated using chewBBACA with a predefined *A. baumannii* schema, excluding paralogues by only including loci present in ≥95% of genomes. Phylogeny trees of the isolates were constructed using Interactive Tree of Life (https://itol.embl.de/itol.cgi). Plasmids were identified using the MOB Suite SoluPlatform (https://platform.solugenomics.com/w/solu) for mobilizable and non-mobilizable plasmids and their contents [36].

Genomic islands were predicted using IslandViewer 4 (http://www.pathogenomics.sfu.ca/islandviewer/), which integrates IslandPath-DIMOB, SIGI-HMM and IslandPick prediction methods. Predicted islands were visualized on circular genome maps and examined for the presence of ARGs, virulence factors and mobile element–associated proteins.

To determine the genetic relatedness of the *A. baumannii* isolates, SNP distances were calculated using the BacWGSTdb 2.0 database (http://bacdb.cn/BacWGSTdb/ [36]). This tool facilitated a comprehensive comparison by aligning the WGS data against *A. baumannii* AYE (ST231) (GenBank accession: CU459141), subsequently generating an SNP distance matrix to quantify the genetic divergence between strains.

## Results

### Prevalence of *A. baumannii* in clinical and environmental reservoirs

A total of 3,235 One Health samples were taken between March 2023 and January 2025. An initial 88 isolates were identified as * A. baumannii* based on biochemical tests; however, only 62 were *bla*_OXA51-like_ positive, with a prevalence of 4.1% for clinical samples, 2.0% for hospital environmental samples, 0.4% for non-hospital environmental samples and 0.5% for poultry dropping samples (Table 1). Isolates with the S prefix were sequenced at the Robert Koch Institute, while isolates with the N prefix were sequenced at the London School of Hygiene and Tropical Medicine. The prefixes were used solely for laboratory tracking purposes and do not indicate biological or epidemiological differences between isolates.

Of the 580 clinical samples collected from AAKTH, MMSH, MAWTH and IDH, including urine (66), sputum (441) and wound swabs (73), 24 *A*. *baumannii* isolates were obtained, amounting to a prevalence of 4.1% (Table 2). These specifically comprised 18 from sputum (4.1%) and 6 from urine (9.1%), while none were recovered from wound samples (0%) (Table 2).

**Table 2.** Distribution of *A. baumannii* isolates from clinical samples across four hospitals

Hospitals	Wound	Urine	Sputum	Total (%)
AKTH	0/28	2/25	5/141	7/194 (3.6%)
MMSH	0/25	0/30	6/191	6/246 (2.4%)
MAWTH	0/20	4/11	6/82	10/113 (8.8%)
IDH	0/0	0/0	1/27	1/27 (3.7%)
Total	0/73	6/66	18/441	24/580 (4.1%)

Of the total 1,605 hospital environment samples collected across AKTH, MMSH and MAWTH, 33 samples (2.0%) contained * A. baumannii* (Table 3). High levels of *A. baumannii* isolation were found on bed rails (12%), suction tubes (7.7%) and ward floors (4.6%) (Table 3). Low-level isolation of *A. baumannii* was observed for bed surfaces (3.7%), toilet floors (0.9%), bedside tables (0.9%), soil samples (0.6%) and biomedical wastes (0.8%). No *A. baumannii* was recovered from ward door handles, toilet door handles, toilet sinks, air samples, thermometers, sewage or dumpsite soil (Table 3).

**Table 3.** Site-specific recovery of *A. baumannii* from hospital environmental samples

Site	AKTH	MMSH	MAWTH	No. of positive (%)
Bed rails	5/45	5/39	4/24	14/108 (12.0%)
Bed surfaces	2/45	0/39	2/24	4/108 (3.7%)
Bedside tables	1/45	0/39	0/24	1/108 (0.9%)
Ward floors	3/45	1/39	1/24	5/108 (4.6%)
Ward door handles	0/45	0/39	0/24	0/108 (0.0%)
Toilet door handles	0/45	0/39	0/24	0/108 (0.0%)
Toilet floor	0/45	0/39	1/24	1/108 (0.9%)
Toilet sinks	0/45	0/39	0/24	0/108 (0.0%)
Ward air samples	0/45	0/39	0/40	0/128 (0.0%)
Thermometers	0/30	0/30	0/30	0/90 (0.0%)
Suction tubes	0/24	0/27	6/26	6/77 (7.7%)
Sewage	0/40	0/40	0/40	0/120 (0.0%)
Soil	1/50	0/50	0/50	1/150 (0.6%)
Biomedical wastes	1/40	0/40	0/40	1/120 (0.8%)
Dumpsite soil	0/20	0/20	0/20	0/60 (0.0%)
Total	13/609	6/558	14/438	33/1,605 (2.1%)

Only 2 (0.3%) of the 530 non-hospital environmental samples collected yielded *A. baumannii* isolates (1 from community sewage and 1 from a municipal dumpsite). No *A. baumannii* isolates were recovered from soil or air samples from non-hospital environments (Table 3). Similarly, poultry-associated samples showed a low recovery rate, with 3 isolates (0.5%) obtained from 520 poultry droppings, 2 of which originated from commercial farms, while 1 was obtained from a poultry market (Table 4).

**Table 4.** Prevalence of *A. baumannii* in non-hospital environmental and poultry-associated samples

Category	Site	No. of positive (%)
**Non-hospital environmental samples**	Sewage	1/120 (0.8%)
	Soil	0/120 (0.0%)
	Municipal dumpsite	1/120 (0.8%)
	Dumpsite air	0/70 (0.0%)
	Dumpsite soil	0/100 (0.0%)
	**Total**	**2/530** (**0.3%**)
**Poultry droppings**	Farms	2/400 (0.5%)
	Markets	1/120 (0.8%)
	**Total**	**3/520** (**0.5%**)

### Antimicrobial susceptibility profiles

**Fig. 1.**
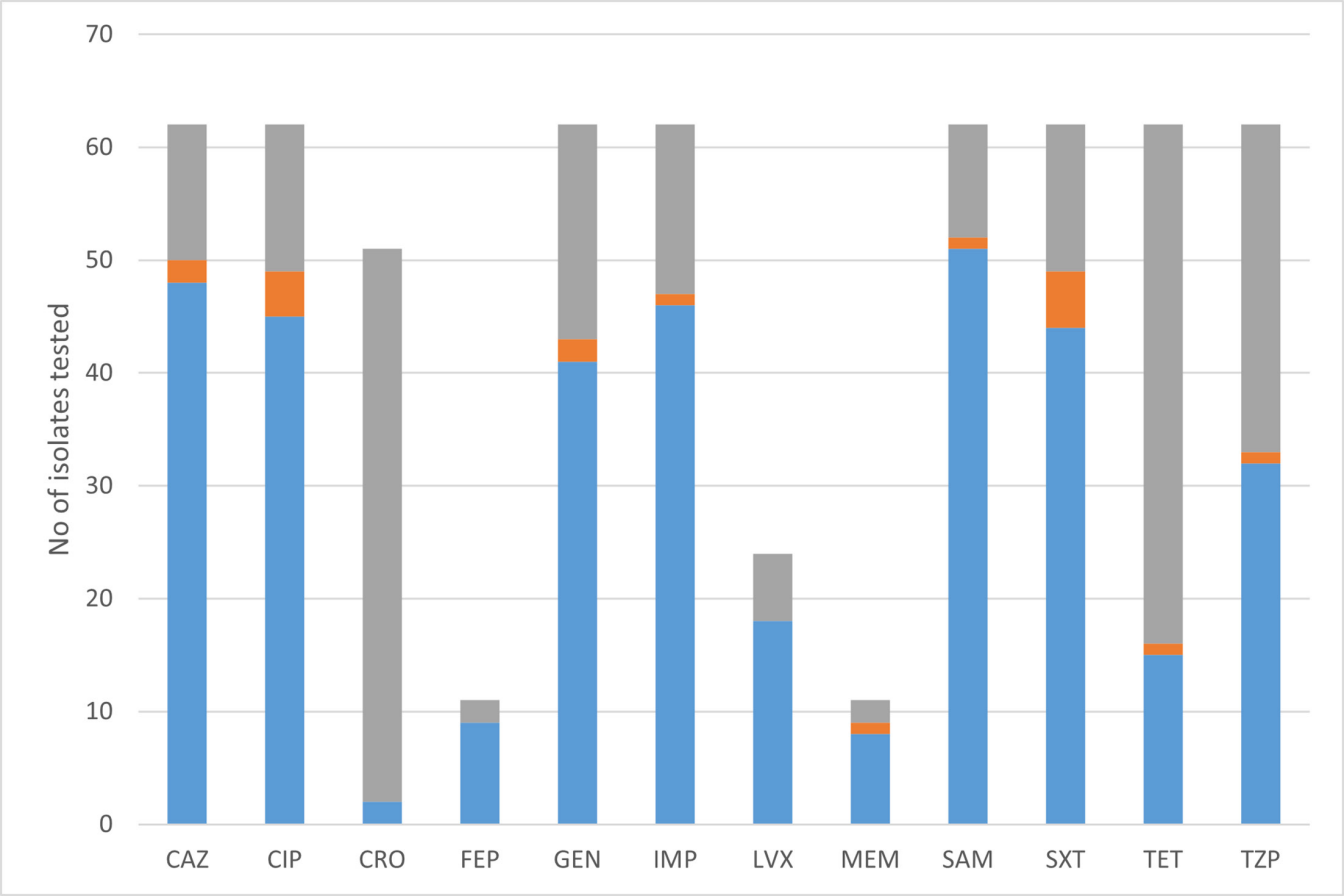
Antibiotic susceptibility profiles of 62 *A*. *baumannii* isolates obtained from human, animal and environmental sources. Isolates were classified as resistant (grey), intermediate (orange) and susceptible (blue) using CLSI disc diffusion guidelines. CAZ, ceftazidime; CIP, ciprofloxacin; CRO, ceftriaxone; FEP, cefepime; GEN, gentamicin; IMP, imipenem; LVX, levofloxacin; MEM, meropenem; SAM, ampicillin–sulbactam; SXT, sulfamethoxazole-trimethoprim; TET, tetracycline; TZP, piperacillin–tazobactam.

Antimicrobial susceptibility testing of the 62 confirmed *A. baumannii* isolates revealed notable variability in resistance patterns (Fig. 1). Four isolates (6.4%) recovered from a burns ward bed surface (N16), a medical ward bed rail (N10), a surgical ward floor (N13) and a municipal dumpsite (N9) exhibited extreme drug resistance, showing complete resistance to all antibiotics tested (File S1). In contrast, most other isolates showed comparatively lower resistance levels, with samples obtained from clinical specimens (*n*=21), hospital environmental surfaces (*n*=9), poultry droppings (*n*=2) and non-hospital environmental samples exhibiting resistance to only two or fewer antibiotic classes (File S1).

Resistance to third-generation cephalosporins varied substantially. Twelve isolates (12/62, 19.3%) were resistant to ceftazidime, with 2 (2/12, 17%) originating from urine samples (S6a, S312), while 9 (9/12, 75%) were recovered from hospital environmental surfaces (Fig. 1 and File S1). In contrast, resistance to ceftriaxone was the most prevalent (49/51, 96%). Ceftriaxone-resistant isolates were recovered from soil (S4), incinerator surfaces (S5), poultry droppings (N21 and N25) and non-hospital environmental sites (S6) (File S1).

Resistance to *β*-lactam/*β*-lactamase inhibitor combinations was also variable. Piperacillin-tazobactam resistance was relatively high (29/62, 46.7%), with 18 (29.0%) isolates recovered from hospital environmental surfaces, 9 (14.5%) from clinical specimens and 2 (3.2%) from poultry droppings. In contrast, ampicillin–sulbactam resistance was notably lower (10/62, 16.1%) (File S1).

MDR isolates were defined as exhibiting non-susceptibility to at least one agent in three or more antimicrobial classes. Isolates were considered extensively drug resistant (XDR) if they were non-susceptible to at least one agent in all but two or fewer antimicrobial classes (i.e. bacterial isolates remain susceptible to only one or two categories), while pan-drug-resistant isolates were those exhibiting non-susceptibility to all agents in all antimicrobial classes [37].

### Whole-genome analysis

Of the 62 recovered isolates, 27 were selected for WGS based on their MDR or XDR phenotypes, while ensuring representation across different sample sources, including clinical, hospital environmental, community and animal-associated samples. Selection was guided by the study objectives and available sequencing resources (accession PRJNA1367586). Multidrug-resistant and non-redundant isolates with high-quality DNA were prioritized to ensure reliable genome assembly (File S1). The assembled genome sizes ranged from 3,550,644 to 4,070,407 bp, with completeness values between 99.82% and 100%, contamination levels from 0% to 2.23% and G+C content between 38.90 and 39.17 mol%.

Thirteen *bla*_OXA-51_ variants were detected among the 27 isolates (48.1%), with *bla*_OXA-120_ being most frequent (*n*=7, 25.9%) (Table 5). Six aminoglycoside-encoding resistance genes [*aac*(*3’*)*-IId*, *ant*(*2’*)*-IIa*, *ant*(*3’*)*-IIa*, *aph*(*3’*)*-Ib*, *aph*(*3’*)*-VIa* and *aph*(*6’*)*-Id*] were detected with varied patterns among the strains (Table 5). *aac*(*3’*)*-IId* was only detected in N15, *ant*(*2’*)*-Ia* in three isolates (N9, N10 and N12), *ant*(*2’*)*-IIa* in five isolates (N9, N10, N13, N14 and N16), *aph*(*3’*)*-Ib* in N14 and N32 and *aph*(*3’*)*-VIa* in four isolates (N9, N10, N13 and N16), as *ant*(*3’*)*-IIa* was detected in all isolates.

**Table 5.** Distribution of *bla*_OXA_ variants among environmental and clinical *A*. *baumannii* isolates

*bla*_OXA-51_ variants	No. of isolates	Isolate IDs	Source of isolates
*bla*_OXA-67_	2	N3 N15	Bed rail Hospital dumpsite
*bla*_OXA-68_	1	N14	Bed rail
*bla*_OXA-90_	2	N10 N19	Bed rail Bed surface
*bla*_OXA-91_	1	N12	Ward floor
*bla*_OXA-120_	7	N1 N2 N4 N6 N8 N21 S5	Toilet floor Bed rail Bed rail Bed rail Urine Poultry Incinerator
*bla*_OXA-180_	1	N28	Poultry dropping
*bla*_OXA-378_	4	N9 N13 N16 N17	Municipal dumpsite Ward floor Bed surface Suction tube
*bla*_OXA-430_	1	N18	Suction tube
*bla*_OXA-707_	1	N7 S4	Ward floor Soil
*bla*_OXA-715_	1	N33	Ward floor
*bla*_OXA-699_	2	N5 N24	Bed rail Poultry
*bla*_OXA-1328_	1	S6	Community sewage
bla_OXA-51_	1	N25	Poultry dropping
***bla*_OXA-58_ variant**	**No. of isolates**	**Isolate IDs**	**Source of isolates**
*bla*_OXA-420_	1	N32	Bed rail

In contrast, macrolide encoding resistant genes (*msrE* and *mphE*) were detected in nine strains: N9, N10, N12, N13, N14, N15, N16, N19 and N32 (Table 6). Two sulphonamide encoding resistant genes (*sul2* and *dfrA20*) were identified in eight isolates (N3, N9, N12, N13, N14, N15, N16 and N32) and four isolates (N9, N14, N16 and N32), respectively. Only six isolates harbour tetracycline encoding resistant genes [*tet*(*B*) and *tet*(*39*)]. Aside from the N9 isolate obtained from the municipal dumpsite, none of the isolates from environmental sources (S4, S5 and S6) carried any antibiotic resistance genes (File S3).

**Table 6.** Antibiotic resistance genes detected among *A. baumannii* isolates from this study

Antibiotic class	Genes detected	No. of positive isolates	Isolate IDs
Aminoglycosides	*ant(3’)-IIa*	27	All isolates
	*aac(3’)-IId*	1	N15
	*ant(2’)-Ia*	3	N9, N10, N12
	*ant(2’)-IIa*	5	N9, N10, N13, N14, N16
	*aph(3’)-Ib*	2	N14, N32
	*aph(3’)-Via*	4	N9, N10, N13, N16
	*aph(6)-Id*	2	N14, N32
	*bla*_ADC-2_	1	N33
	*bla*_ADC-32_	6	N5, N9, N13, N15, N16, N24
	*bla*_ADC-52_	1	N10
	*bla*_ADC-76_	3	N3, N14, N32
	*bla*_ADC-155_	1	N17
	*bla*_ADC-156_	8	N1, N2, N4, N6, N8, N18, N21, N25
	*bla*_ADC-203_	1	N7
	*bla*_ADC-158_	1	N28
Macrolides	*msrE*, *mphE*	9	N9, N10, N12, N13, N14, N15, N16, N19, N32
Sulphonamides	*sul2*	8	N3, N9, N12, N13, N14, N15, N16, N32
	*dfrA20*	4	N9, N14, N16, N32
Tetracyclines	*tet(B)*	3	N3, N12, N14
	*tet(39)*	3	N3, N10, N15
Carbapenems	*bla_NDM-1_*	6	N9, N10, N13, N15, N16, N19

MLST analysis using Oxford and Pasteur schemes identified 25 sequence types (STs) in total. Seven novel STs were identified using both schemes that include ST2858^pas^ (N3 and N15), ST3316^pas^ (N5 and N24), ST3318^pas^ (N33), ST3593^oxf^ (N3), ST3599^oxf^ (N7), ST3594^oxf^ (N15) and ST3595^oxf^ (N18). Other known STs detected included ST78^pas^/ST2830^oxf^ (N10), ST10^pas^/ST585^oxf^ (N14, N32), ST821^pas^/ST2146^oxf^ (N9, N13 and N19), ST40^pas^/ST427^oxf^ (N17), ST132^pas^/ST29d8e^oxf^ (N18), ST164^pas^/ST1418^oxf^ (N12), ST625^pas^/ST860^oxf^ (N19), ST216^pas^/ST2955d^oxf^ (N25) and ST459/ST27385^oxf^ (N4, N6). Only three of the isolates belong to two out of the nine recognized ICs. Isolate N10 belongs to IC6 (ST78^pas^/ST2830^oxf^), and isolates N14 and N32 belong to IC8 (ST10^pas^/ST585^oxf^) (Table 7). Three isolates obtained from the hospital burn ward, municipal dumpsite and sewage belonged to ST821 (Pasteur scheme), a lineage which has been previously detected in the UK (ID10089), Pakistan (ID11599: ID11632), Germany (ID: 3394) and Nigeria (ID8425) [10].

**Table 7.** MLST profiles and clonal lineages of *A. baumannii* isolates identified in this study

Category	STs	Isolate IDs	Notes
Novel STs (Pasteur scheme)	ST2858^pas^ ST3316^pas^ ST3318^pas^	N3, N15 N5, N24 N33	– – –
Novel STs (Oxford scheme)	ST3593^oxf^ ST3599^oxf^ ST3594^oxf^ ST3595^oxf^	N3 N7 N15 N18	– – – –
Known STs	ST78^pas^/ST2830^oxf^	N10	IC6
	ST10^pas^/ST585^oxf^	N14, N32	IC8
	ST821^pas^/ST2146^oxf^	N9, N13, N16	Resistant to all antibiotics tested
	ST40^pas^/ST427^oxf^ ST3534 ^oxf^ ST2122^pas^ ST2790^pas^/ST8276^oxf^	N17 N5, N24 N7 N1, N2, N8, N21	– – – –
	ST132^pas^/ST29d8e^oxf^	N18	–
	ST164^pas^/ST1418^oxf^	N12	–
	ST625^pas^/ST860^oxf^	N19	–
	ST216^pas^/ST2955^oxf^	N25	–
	ST459^pas^/ST27385^oxf^ ST2684 ^pas^/ST942^oxf^ ST2132^pas^/ST3122^oxf^ ST2790 ^pas^/ST3457^oxf^ ST1211^pas^/ST3122^oxf^	N4, N6 N28 S4 S5 S6	– – – – –

Allelic analysis revealed that N3 possessed a novel *gyrB* allele but shared five alleles with ST3457^oxf^; N8, N1, N2 and N28 also shared exact *gyrB* alleles with ST3457^oxf^. N4 and N6 carried novel *gpi* and *gdhB* alleles, respectively, but shared five alleles with ST717^oxf^, while N7 harboured novel *gltA*, *gdhB*, *rpoD* and *recA* alleles. N25 harboured novel *gpi* and *rpoD* alleles and shared five alleles with ST955^oxf^. N33 carried novel *gdhB* and *cpn60* alleles and exhibited mismatches with ST1203^pas^. Isolates N10, N18 and N15 each displayed single-locus mismatches with ST2830^oxf^, ST75^oxf^ and other existing STs (File S2).

Pairwise SNP distances among isolates ranged from 5 to ~45,000 SNPs. Very close genomic relationships were observed between N1 and N8 (5 SNPs), N9 and N13 (5 SNPs), N13 and N16 (12 SNPs), N16 and N9 (17 SNPs), N14 and N32 (22 SNPs) and N4 and N6 (26 SNPs) (Table 8; Fig. S1). To put this into context, existing ST821 genomes from public databases were included in the SNP matrix. This comparison revealed notable genetic divergence between the database ST821 isolates and those from this study. For example, the ST821 isolate OUT6-2_S19-Ibadan and isolate N9 differed by 2,630 SNPs, highlighting their distant genomic relatedness despite sharing the same ST (Fig. S1).

**Table 8.** Pairwise SNP distances and epidemiological interpretation of isolates with SNP distances (5–135)

Isolate pair	Source(s)	SNP distance	Interpretation (based on ECDC/UKHSA)	Recommended action
N1 vs N8	Female medical ward toilet floor vs dialysis patient’s urine	5	Clonal (≤10 SNPs), consistent with recent or ongoing transmission between hospital environment and patient.	Immediate IPC review; contact tracing; screening of linked patients and environmental sites.
N5 vs N24	Paediatric unit bed rail vs poultry dropping	135	Unrelated (>100 SNPs), indicating no evidence of recent transmission.	No outbreak link; routine surveillance only.
N32 vs N14	Paediatric unit bed rail vs male medical ward floor	22	Related but not clonal (>20 SNPs), suggesting persistence of a common lineage within the hospital rather than recent direct transmission.	Review environmental persistence and cleaning schedules; assess temporal overlap.
N4 vs N6	Paediatric unit bed rail vs female surgical ward bed rail	26	Borderline related (>20 SNPs); recent common ancestry possible, but genomics alone does not support a confirmed outbreak.	Integrate epidemiologic data, sampling dates and ARG/plasmid profiles.
N9 vs N13	Municipal dumpsite vs hospital floor	5	Clonal (≤10 SNPs), indicating recent transmission or shared contamination source linking community environment and hospital surfaces.	Investigate waste-handling routes; strengthen environmental IPC and entry-point controls.
N9 vs N16	Municipal dumpsite vs bed surface	17	Closely related (11–20 SNPs), consistent with circulation of the same strain across connected external and hospital reservoirs.	Investigate waste-handling routes; include environmental monitoring in IPC plans.
N13 vs N16	Hospital floor vs bed surface	12	Closely related (11–20 SNPs), suggesting environmental dissemination within the hospital.	Review environmental persistence and cleaning schedules; assess temporal overlap.

ECDC, European Centre for Disease Prevention and Control; UKHSA, UK Health Security Agency.

**Fig. 2.**
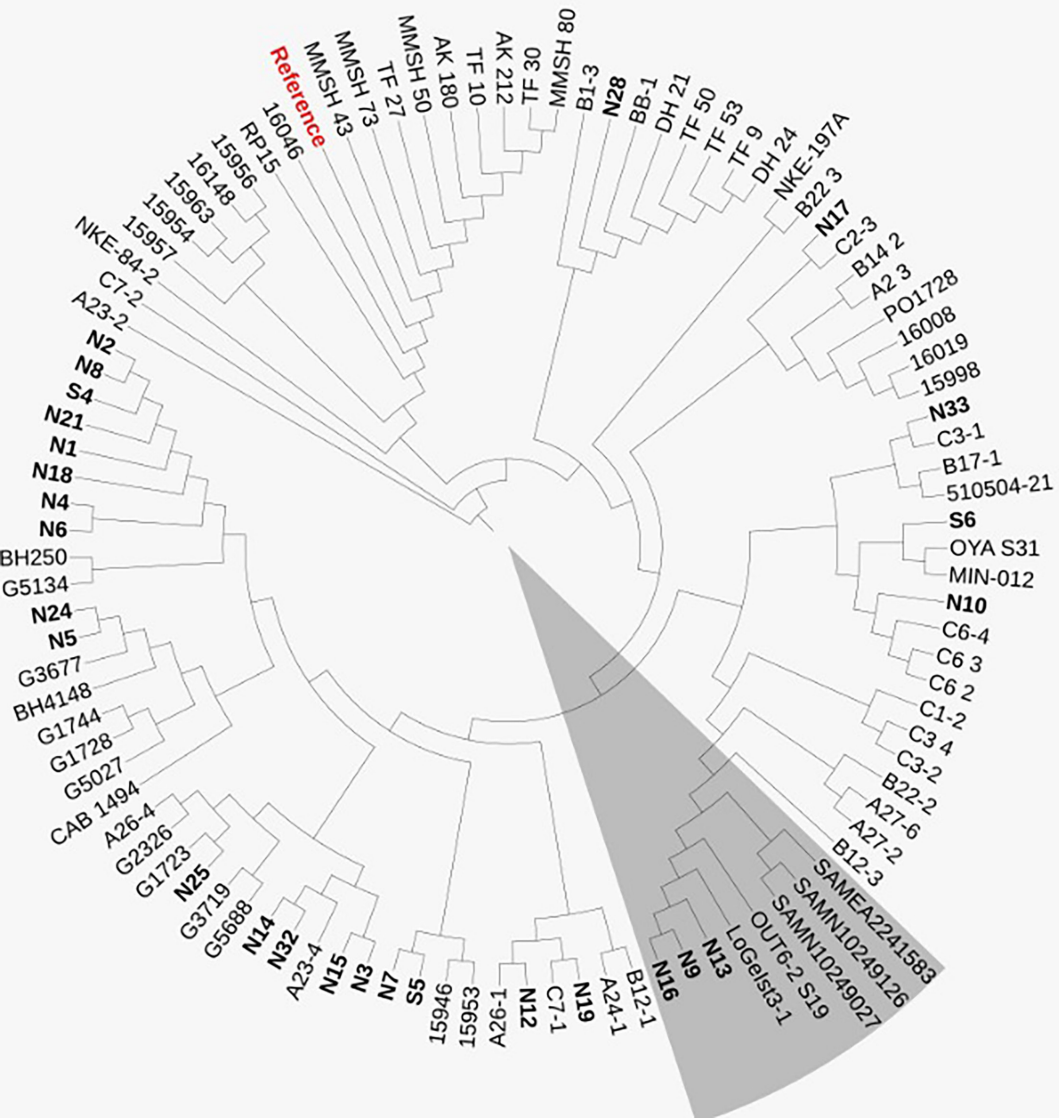
Maximum-likelihood phylogenetic analysis of *A. baumannii* isolates. Phylogenetic relationship of *A. baumannii* isolates from this study (shown in bold black) clustered with a diverse set of Nigerian and international reference genomes. The tree was constructed based on core-genome SNPs to show clonal dissemination. A prominent clade representing the emerging high-risk lineage ST821 (grey cone shape), showing a transcontinental distribution of *A. baumannii* that includes isolates from this study (**N9, N13 and N16**), previous Nigerian reports (OUT6-2 S19) [10] and international strains from the UK (LoGelst3-1), Germany (SAMEAA2241583) and Pakistan (SAMN10249126 and SAMN10249127). Metadata for the *A. baumannii* isolates from this study, Nigerian isolates deposited in NCBI from previous studies and other ST821 isolates presented in the circular tree are available in the supplementary section (File S3).

The core genome phylogenetic analysis reveals a notable transcontinental distribution within the ST821 lineage, characterized by a tight cluster of isolates from diverse geographical origins (Fig. 2). Specifically, this clade incorporates contemporary isolates from the current study (N9, N13 and N16) alongside previously sequenced strains from the UK (LoGelst3-1), Nigeria (OUT6-2 S19), Germany (SAMEA2241583) and Pakistan (SAMN10249126 and SAMN10249127). The clustering of the UK, German and Pakistani isolates with Nigerian isolates indicates that ST821 is not merely a localized variant but an emerging high-risk clone with international dissemination potential. The high degree of genetic conservation between the isolates from our study and those from disparate geographic regions indicates a recent common ancestor and highlights the role of international travel and trade in the global spread of MDR *A. baumannii* (Fig. S1).

**Fig. 3.**
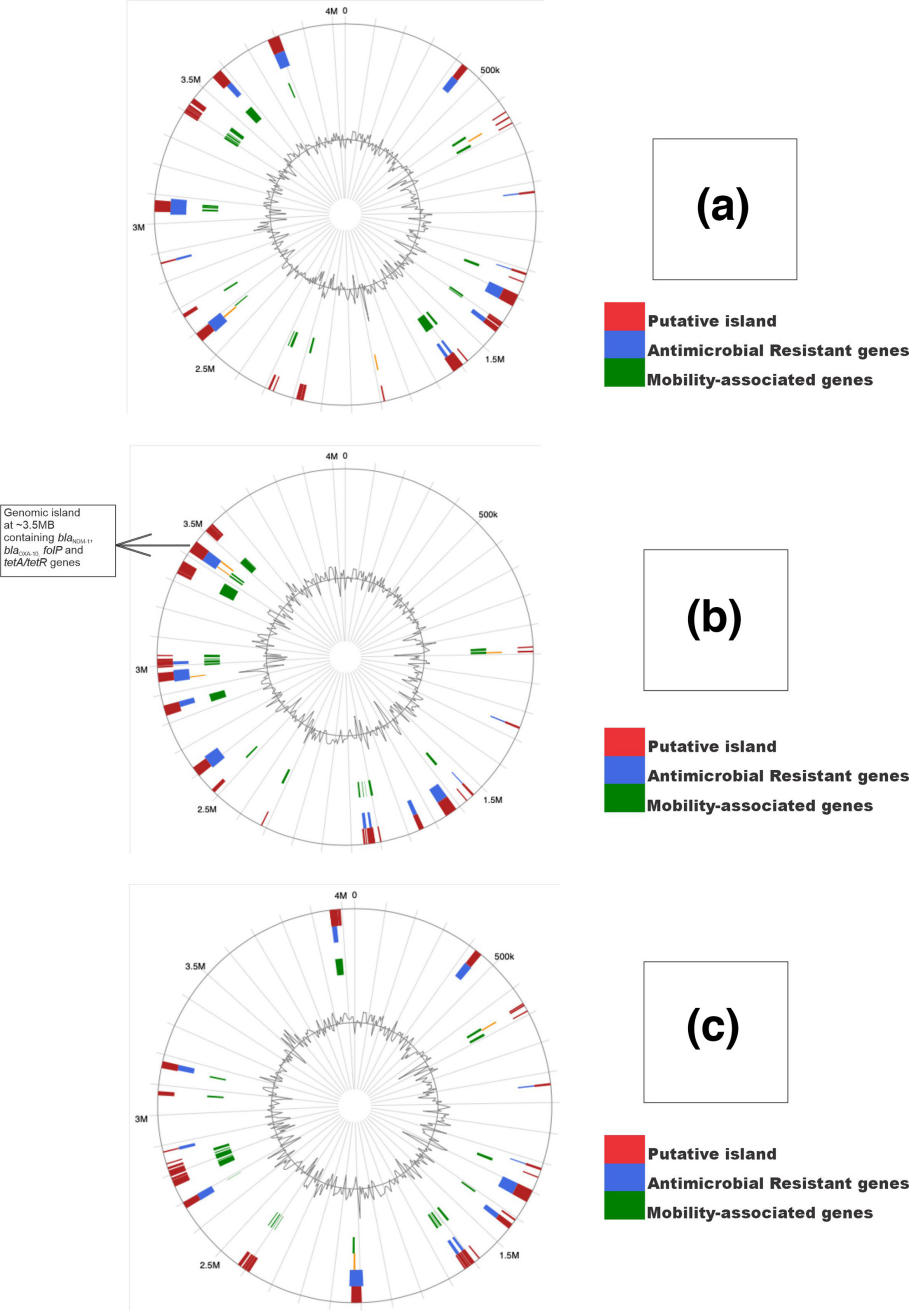
Circular genome maps of extreme drug-resistant *A. baumannii*. Maps of isolates N9 (**a**), N13 (**b**) and N16 (**c**) illustrate the distribution of genomic islands, antimicrobial resistance determinants and mobile genetic elements. The outermost ring represents annotated genomic features, including predicted genomic islands (red), ARGs (blue) and mobility-associated elements (green). Inner rings show coding sequences, repeat regions and GC skew, and the central plot indicates GC content variation across the genome.

Genomic islands were detected in N9, N13 and N16 (Fig. 3), which a comparative analysis revealed two distinct but complementary loci of clinical and biological significance. The island located at ~3.5 Mb for N13 primarily contains multiple ARGs, including *bla*_NDM-1_, *bla*_OXA-10_ (conferring carbapenem resistance), aminoglycoside-modifying enzyme genes, *folP* (sulphonamide resistance) and *tetA/tetR* (dfr-type tetracycline resistance), and thereby constitutes an MDR genomic island. This genomic island also carries genes encoding several toxin–antitoxin systems (RelBE, HicAB, YafO–YafN and VbhTA); multiple mobility-associated factors, including transposases, integrases, HNH endonucleases and DNA repair; and mutagenesis enzymes such as UmuC/UmuD and DNA adenine methylases. Phage-associated genes, including capsid, tail and terminase proteins, as well as Mu-like and P2-like prophage elements, were also identified. Additional genes related to transport, metabolism and stress response, including components of the Tat secretion system and TonB-dependent receptors, were identified within the genomic islands.

In contrast, the island at ~1.8 Mb displays a broader functional profile, combining aminoglycoside resistance genes (*aac*, *aphA*) and tetracycline resistance determinants (*tetA/tetR*) with stress adaptation factors (*dinB*, *cueR* and *trxC*) and metabolic genes (*cysK*, *coaBC*, *moeB* and *mnmC*). While the 3.5 Mb island appears to be a focused resistance hotspot with strong clinical implications due to *bla*NDM-1, the 1.8 Mb island represents a more multifunctional adaptation island, integrating resistance to some antibiotics with stress tolerance and metabolic versatility.

## Discussion

The study provides a comprehensive One Health genomic surveillance of *A. baumannii* across clinical samples, hospital environments, non-hospital environments and animal sources in Kano, Nigeria. Out of the 3,245 samples analysed, 62 contained * A. baumannii* isolates of which 27 were sequenced and underwent molecular analysis, including typing. Our findings indicate the hospital environment as a key reservoir for *A. baumannii*, while also revealing the emergence and environmental dissemination of a high-risk drug-resistant lineage (ST821) across hospital and community settings.

The highest recovery rates of *A. baumannii* were from clinical samples (4.1%). Lower rates were recovered from hospital environmental samples (2.1%), non-hospital environmental samples (0.4%) and poultry dropping samples (0.8%) consistent with reports from other low and middle income country (LMIC) settings, where *A. baumannii* is often under detected or largely isolated from targeted clinical or surveillance studies rather than broad environmental and clinical sampling. The disproportionately low recovery of *A. baumannii* from environmental and animal sources suggests that while this species is a formidable clinical pathogen, it may occupy a narrower ecological niche in Kano than previously hypothesized. This recovery gap likely reflects a combination of intense microbial competition in non-clinical environments and the potential transition of isolates into a viable but non-culturable state under environmental stress.

A systematic review of hospital-acquired *A. baumannii* highlights the paucity of data in Africa and generally very low incidence (incidences between 0.85 and 5.6 cases per 1,000 patients) [38] . In Nigeria, non-ICU hospital surveillance studies have reported overall *A. baumannii* prevalence rates of 2.0–6.7%, reflecting comparatively modest recovery beyond outbreak clusters [39]. Similarly, a meta-analysis from Ethiopia found a pooled *A. baumannii* prevalence of ~4%, supporting the observation that overall detection rates in routine clinical settings – without direct focus on MDR or epidemic isolates – can be relatively low [40].

The highest prevalence in this study was observed among clinical samples (9.5%), with isolates predominantly recovered from sputum, reflecting the well-established association of *A. baumannii* with lower respiratory tract infections [41,42]. Analysis of 150 patient samples in a Nigerian hospital study reported that the majority of *A. baumannii* isolates were obtained from sputum, which aligns with findings in this study [43]. The absence of isolates from wound samples contrasts with reports from intensive care and burn-unit settings, where *A. baumannii* is frequently recovered from wound infections [44,46]. However, this may reflect differences in patient populations, sampling practices or infection control measures rather than true absence of the organism [47].

Hospital environmental sampling revealed contamination, with *A. baumannii* recovered from 2.1% of surfaces, particularly high-touch and moisture-associated sites such as bed rails, suction tubes and ward floors. These findings reflect the ability of the bacterium to resist desiccation and persist on abiotic surfaces, facilitating indirect transmission and infection [48]. The predominance of isolates from bed rails and suction equipment highlights critical lapses in environmental cleaning and device decontamination, reinforcing the concept that hospital environments are active reservoirs of potential infection. In contrast, recovery from non-hospital environments and poultry droppings was low (<1%), showing that *A. baumannii* was present in community and animal-associated niches but isolated less frequently than in healthcare settings in this study.

Antimicrobial susceptibility testing (AST) revealed marked heterogeneity, with most isolates from all sampling sites exhibiting limited resistance. Nonetheless, the detection of MDR isolates (6.3%) is of major concern. The highly resistant strains, including MDR (N12, N15 and N19) and XDR isolates (N9, N10, N13 and N16), were recovered from both hospital environments and community-associated sites, including a municipal dumpsite, indicating that resistant strains are no longer confined to clinical settings and can be found in environmental external sites. Furthermore, the observed resistance to ceftriaxone, tetracycline and piperacillin–tazobactam is consistent with trends reported globally and further underscores the already narrow therapeutic options available for treatment of these isolates [49].

WGS of selected isolates revealed extensive genetic diversity, including 16 bla_OXA-51_ variants and a high proportion of novel STs, highlighting ongoing local evolution. The identification of ST821 as an XDR lineage across municipal dumpsites, hospital floors and bed surfaces represents one of the most significant findings of this study. While ST821 has previously been reported in Nigeria and other countries as largely susceptible [15], our data demonstrate the emergence of a highly resistant variant harbouring *bla*_NDM-1_ and multiple resistance islands.

Pairwise SNP analysis showed close relatedness among the local ST821 isolates (5–17 SNPs), suggesting recent divergence and local clonal expansion. Contrarily, there were large SNP distances between these isolates and international ST821 strains, indicating independent evolutionary paths rather than recent importation. The marked contrast between the XDR Nigerian ST821 isolates described in this study and previously reported susceptible ST821 strains from Nigeria (Ibadan) and other regions suggests recent local adaptation under selective pressure. In many LMIC settings, frequent empirical antibiotic use, inconsistent antimicrobial stewardship and limited diagnostic-guided prescribing practices may create conditions that favour the emergence of resistant lineages. In addition, acquisition of multiple resistance determinants through transposons, integrons or resistance islands may have accelerated the evolution of XDR ST821. The recovery of near-clonal ST821 isolates across municipal waste and hospital surfaces suggests that environmental reservoirs may play a direct role in the establishment and dissemination of multidrug-resistant *A. baumannii* in Kano.

The coexistence of extensive resistance determinants and complete virulence gene repertoires in some isolates, including environmental strains, further raises concern about their potential to cause localized infection outbreaks. The presence of multiple insertion sequences (ISs) associated with resistance expression, together with toxin–antitoxin systems and prophage elements, suggests enhanced genomic stability and adaptability, potentially facilitating long-term survivability under antimicrobial and environmental stress.

Importantly, resistance determinants were largely chromosomally encoded, as evidenced by the absence of mobile plasmids in most isolates.

All XDR isolates (N9, N13 and N16) harboured CRISPR repeat-spacer arrays (File S4). However, no cas genes were detected, indicating that these systems are likely non-functional or degenerate remnants lacking adaptive immunity [50]. Despite the presence of CRISPR loci, no plasmids were identified across the genomes, suggesting that plasmid absence is not attributable to CRISPR-mediated interference. Given the XDR phenotype of isolates, AMR is likely mediated by chromosomally encoded or stably integrated mobile genetic elements rather than plasmid-borne determinants [51,53]. Collectively, these findings suggest a clonal adaptation strategy in which resistance is maintained independently of plasmid carriage, while CRISPR remnants persist as degenerated CRISPR loci lacking associated cas genes.

Instead, AMR was predominantly mediated by chromosomal resistance islands, ISs and composite transposons, underscoring the central role of chromosomal plasticity in *A. baumannii* adaptation [54]. This genomic architecture is characteristic of * A. baumannii*, where resistance genes are frequently embedded within chromosomal AbaR-like islands and amplified or mobilized by IS elements rather than carried on plasmids [55,57]. Such arrangements facilitate stable vertical inheritance of resistance traits and support long-term persistence under antimicrobial pressure [56,58, 59].

This is important in clinical settings and One Health because the chromosomal integration of AbaR islands ensures the permanent fixation of MDR traits, allowing high-risk *A. baumannii* clones to persist in clinical and environmental niches without the fitness costs of plasmid maintenance. This stable genetic architecture facilitates the reliable vertical inheritance of complex resistance profiles, ensuring that entire bacterial clades remain multidrug-resistant even in the absence of direct antimicrobial pressure.

Within a One Health framework, these constitutively conserved clades act as resilient reservoirs that survive hospital disinfection and environmental shifts, facilitating global dissemination across humans, animals and water systems. Furthermore, the stable island serves as a genomic scaffold for the further accumulation of resistance genes, driving the evolution of extreme drug-resistant strains. Consequently, identifying these vertically transmitted lineages is crucial for effective infection control.

The observation in this study is consistent with our previous report from the same study area, which similarly identified an absence of plasmids among *A. baumannii* isolates alongside the presence of multiple, often truncated, IS elements [12,17]. The recurrence of truncated and disrupted ISs suggests ongoing genomic rearrangements and historical transposition events, reflecting continuous local adaptation rather than recent acquisition of resistance via horizontal plasmid transfer. Collectively, these observations support the view that chromosomal remodelling rather than plasmid-mediated dissemination is the dominant mechanism driving multidrug and extreme drug resistance in *A. baumannii* circulating within both clinical and environmental niches in this setting.

Overall, our findings show that *A. baumannii* circulating in Kano, Nigeria, is characterized by high genetic diversity, environmental circulation and the emergence of a locally evolved high-risk ST821 lineage that spans across hospital and community settings. The findings stress the urgent need for integrated One Health surveillance, reinforced infection prevention and control, rational antimicrobial stewardship and targeted environmental hygiene interventions. Without coordinated action, high-risk hospital-derived clones may continue to seed community and environmental reservoirs, further perpetuating antimicrobial resistance and difficult-to-treat infection.

## Supplementary material

10.1099/mgen.0.001768 Supplementary Material 1.

10.1099/mgen.0.001768 Supplementary Material 2.

## Abbreviations

AKTH: Aminu Kano Teaching Hospital
AMR: antimicrobial resistance
ARGs: antimicrobial resistance genes
ICs: international clones
IDH: Infectious Diseases Hospital
ISs: insertion sequences
MAWTH: Muhammad Abdullahi Wase Teaching Hospital
MDR: multidrug resistance
MMSH: Murtala Muhammad Specialist Hospital
MSM: mineral salt medium
STs: sequence types
WGS: whole-genome sequencing
XDR: extensively drug resistant
