# Supplementary material for: One Health genomic surveillance identified high-risk carbapenem-resistant ST821 clones of Acinetobacter baumannii in Nigerian clinical and community settings
Source: Microb Genom. 2026 Jun 29;12(6):001768. doi: 10.1099/mgen.0.001768 (PMC13313168; doi:10.1099/mgen.0.001768)
Supplement: Supplementary Material 1. [file mgen-12-01768-s001.pdf]

	LoGelst3-1	N10	N12	N13	N14	N15	N16	N17	N18	N19	N1	N21	N24	N25	N28_	N2	N32	N33	N3	N4	N5	N6	N7	N8	N9	OUT6-2_S19	S4	S5	S6	SAMEA2241583	SAMN10249027	SAMN10249126
LoGelst3-1	0	43967	43928	1387	46511	44329	1375	45274	45199	44550	44781	45015	44449	44323	40854	44972	46500	43821	44183	44125	44426	44127	44875	44778	1388	2309	44924	45055	44131	5340	5585	5577
N10	43967	0	43364	43890	45937	44457	43886	44817	44752	43741	44157	44381	44678	43460	40192	44346	45928	43590	44391	44484	44678	44490	44307	44154	43893	44397	44488	44449	43600	43515	43474	43486
N12	43928	43364	0	43872	45209	43542	43866	42782	43597	42563	43508	43814	43703	42313	39027	43782	45198	43180	43589	42701	43656	42699	43010	43507	43875	44351	43152	43820	43639	43528	43730	43748
N13	1387	43890	43872	0	46363	44316	12	45378	45241	44528	44835	44916	44626	44137	41026	44908	46352	43801	44170	44259	44612	44261	44826	44834	5	2317	44940	44970	44158	5412	5463	5465
N14	46511	45937	45209	46363	0	33088	46359	46596	46361	46495	46027	46079	45928	45156	40225	46091	22	45095	33141	45436	45905	45436	44884	46026	46364	46713	45010	46149	44957	46061	46211	46225
N15	44329	44457	43542	44316	33088	0	44304	45062	45070	44469	44618	44753	43489	34593	39425	44697	33082	44204	1955	43991	43496	43993	43391	44615	44317	44767	43565	44809	44201	43918	44098	44096
N16	1375	43886	43866	12	46359	44304	0	45376	45237	44516	44827	44906	44626	44137	41024	44896	46348	43789	44158	44247	44612	44249	44818	44824	17	2311	44938	44960	44156	5400	5459	5453
N17	45274	44817	42782	45378	46596	45062	45376	0	44347	45935	43592	43895	45034	43955	39322	43836	46582	44913	45095	44218	45015	44218	43797	43589	45379	45843	43909	43943	44800	44832	45167	45167
N18	45199	44752	43597	45241	46361	45070	45237	44347	0	45695	11160	11428	37982	42950	38563	11387	46341	45344	44923	18467	37994	18473	44388	11161	45242	45614	44491	11591	44802	44696	45079	45095
N19	44550	43741	42563	44528	46495	44469	44516	45935	45695	0	45465	45686	45589	44182	40584	45660	46487	43651	44495	44776	45575	44784	45052	45462	44527	44888	45198	45757	44039	44248	44207	44209
N1	44781	44157	43508	44835	46027	44618	44827	43592	11160	45465	0	358	37913	43178	38246	293	46017	44666	44443	19918	37927	19922	43883	5	44834	45190	43992	535	44607	44193	44550	44564
N21	45015	44381	43814	44916	46079	44753	44906	43895	11428	45686	358	0	38211	43269	38590	67	46069	45014	44578	20258	38228	20262	44054	353	44917	45340	44165	189	44790	44512	44691	44703
N24	44449	44678	43703	44626	45928	43489	44626	45034	37982	45589	37913	38211	0	42703	38374	38194	45911	44327	43581	38317	138	38321	44636	37910	44627	44965	44716	38268	44720	44029	44665	44667
N25	44323	43460	42313	44137	45156	34593	44137	43955	42950	44182	43178	43269	42703	0	38553	43224	45143	43803	33898	42518	42693	42520	41782	43177	44140	44724	41896	43296	43942	43892	44043	44043
N28_	40854	40192	39027	41026	40225	39425	41024	39322	38563	40584	38246	38590	38374	38553	0	38531	40205	39776	39439	37686	38378	37708	39233	38243	41025	41447	39124	38629	40080	40477	40839	40841
N2	44972	44346	43782	44908	46091	44697	44896	43836	11387	45660	293	67	38194	43224	38531	0	46081	44953	44522	20207	38201	20211	44017	296	44907	45336	44126	248	44754	44464	44664	44674
N32	46500	45928	45198	46352	22	33082	46348	46582	46341	46487	46017	46069	45911	45143	40205	46081	0	45087	33135	45426	45888	45426	44870	46016	46353	46721	44996	46139	44946	46050	46200	46214
N33	43821	43590	43180	43801	45095	44204	43789	44913	45344	43651	44666	45014	44327	43803	39776	44953	45087	0	44031	44449	44333	44451	43944	44663	43800	44285	44108	45075	43042	43199	43565	43569
N3	44183	44391	43589	44170	33141	1955	44158	45095	44923	44495	44443	44578	43581	33898	39439	44522	33135	44031	0	43976	43588	43978	43346	44440	44171	44606	43509	44634	44168	43771	43957	43961
N4	44125	44484	42701	44259	45436	43991	44247	44218	18467	44776	19918	20258	38317	42518	37686	20207	45426	44449	43976	0	38317	26	44000	19917	44260	44567	44122	20358	44203	43608	44087	44093
N5	44426	44678	43656	44612	45905	43496	44612	45015	37994	45575	37927	38228	138	42693	38378	38201	45888	44333	43588	38317	0	38321	44625	37924	44613	44943	44705	38270	44725	44037	44686	44688
N6	44127	44490	42699	44261	45436	43993	44249	44218	18473	44784	19922	20262	38321	42520	37708	20211	45426	44451	43978	26	38321	0	43998	19921	44262	44569	44120	20362	44207	43610	44089	44095
N7	44875	44307	43010	44826	44884	43391	44818	43797	44388	45052	43883	44054	44636	41782	39233	44017	44870	43944	43346	44000	44625	43998	0	43882	44827	45261	521	44138	43558	44360	44400	44414
N8	44778	44154	43507	44834	46026	44615	44824	43589	11161	45462	5	353	37910	43177	38243	296	46016	44663	44440	19917	37924	19921	43882	0	44833	45187	43991	538	44604	44192	44551	44563
N9	1388	43893	43875	5	46364	44317	17	45379	45242	44527	44834	44917	44627	44140	41025	44907	46353	43800	44171	44260	44613	44262	44827	44833	0	2318	44939	44971	44159	5411	5460	5462
OUT6-2_S19	2309	44397	44351	2317	46713	44767	2311	45843	45614	44888	45190	45340	44965	44724	41447	45336	46721	44285	44606	44567	44943	44569	45261	45187	2318	0	45368	45405	44589	5174	5238	5230
S4	44924	44488	43152	44940	45010	43565	44938	43909	44491	45198	43992	44165	44716	41896	39124	44126	44996	44108	43509	44122	44705	44120	521	43991	44939	45368	0	44208	43485	44441	44500	44504
S5	45055	44449	43820	44970	46149	44809	44960	43943	11591	45757	535	189	38268	43296	38629	248	46139	45075	44634	20358	38270	20362	44138	538	44971	45405	44208	0	44784	44580	44748	44760
S6	44131	43600	43639	44158	44957	44201	44156	44800	44802	44039	44607	44790	44720	43942	40080	44754	44946	43042	44168	44203	44725	44207	43558	44604	44159	44589	43485	44784	0	43861	43913	43907
SAMEA2241583	5340	43515	43528	5412	46061	43918	5400	44832	44696	44248	44193	44512	44029	43892	40477	44464	46050	43199	43771	43608	44037	43610	44360	44192	5411	5174	44441	44580	43861	0	1223	1211
SAMN10249027	5585	43474	43730	5463	46211	44098	5459	45167	45079	44207	44550	44691	44665	44043	40839	44664	46200	43565	43957	44087	44686	44089	44400	44551	5460	5238	44500	44748	43913	1223	0	48
SAMN10249126	5577	43486	43748	5465	46225	44096	5453	45167	45095	44209	44564	44703	44667	44043	40841	44674	46214	43569	43961	44093	44688	44095	44414	44563	5462	5230	44504	44760	43907	1211	48	0

Supplementary figure 1. SNP distance matrix. SNP matrix analysis calculated using BacWGSTdb 2.0 database by aligning all isolates genome data against AYE [ST231] (Genbank accession: CU459141). Additional ST821 references from Nigeria (OUT62_S19), UK (LoGelst3-1), Germany (SAMEA2241583), and Pakistan (SAMN10249126 and SAMN10249127) were included. The closest SNP distancing was observed between N13 and N9 with 5 SNPs.
